# “Le Grand absent Européen”: solidarity in the politics of European integration

**DOI:** 10.1057/s41269-020-00171-7

**Published:** 2020-06-12

**Authors:** Andreas Grimmel

**Affiliations:** Fakultät für Wirtschafts- und Sozialwissenschaften, Sozialwissenschaften, Politikwissenschaft, Universität Hamburg, Allende-Platz 1, 20146 Hamburg, Germany

## Abstract

The appeal for more solidarity is a recurring pattern in political discussions of how to cope with the crises the European Union (EU) confronts. This became most palpable in two major challenges—the Eurozone crisis and the refugee and migrant crisis. However, there is a yawning gap between the rhetorical commitment to solidarity and member states’ practices of solidarity: Even though the EU and its members regularly refer to solidarity as one of their fundamental values, the concept regularly fails to translate into concrete and common action. This lack of solidarity when it comes to problem resolution not only renders solidarity a weak principle within the EU’s political framework; it also precludes more effective crisis management. Drawing on the works of Ludwig Wittgenstein as well as (neo)pragmatism, this paper argues that a language- and practice-based reading can offer a new perspective on solidarity as one of the EU’s fundamental values. It can also explain why solidarity does not play a more vital role in the EU today, especially in times of crisis, when it is most needed.

## Introduction: solidarity as a value and practice

One can tell much about the meaning of a concept by looking at the circumstances in which it is used. This especially applies to the use of the concept of solidarity and the increasing invocation of the term in the recent crises in the European Union (EU)—including the Eurozone and the refugee crises, the crisis in the wake of the COVID-19 pandemic, and the Brexit process. A communication from the Commission to the European Parliament, the Council, the European Economic and Social Committee, and the Committee of the Regions, for example, claims that “[m]ore solidarity will keep Europe together. It provides the necessary unity to cope with current and future crises by holding a strong moral ground.”^1^ However, such calls for solidarity in the EU ring hollow if the concept is not applied in practice, and in contrast to the frequent references to the value of solidarity in political declarations and debates, it has had little impact on the EU’s actual crisis management. The result is a somewhat paradoxical situation in which recalling the concept of solidarity as one of the core values of the European integration process is sharply contrasted by the insight that “[l]e grand absent européen, c’est la solidarité,” as the former president of the European Commission, Jean-Claude Juncker, acknowledged in May 2017.^2^

Today’s disagreement regarding how the concept translates into practice within the EU is even more striking when one considers that solidarity has always been at the center of the EU’s perception of itself (for an overview, see Sangiovanni 2013), most explicitly expressed by Robert Schuman, who claimed already in 1950 that “Europe will not be made all at once, or according to a single plan [, but that it] will be built through concrete achievements which first create a de facto solidarity.”^3^ Interestingly, Schuman was not distinguishing between the individual, collective and the institutional levels when demanding solidarity as the foundation for the new European project that he envisioned. Based on this foundational commitment that was accepted by the members states and that ever since became a leitmotif in the integration process, the concept of solidarity was codified in the Preamble to the Treaty Establishing the European Coal and Steel Community (1951), the Single European Act (1986), the Maastricht Treaty (1992) and the Treaty of Lisbon (2009) (for a detailed discussion on the diverse dimensions of solidarity in EU law, see Domurath, 2013). As such it was demanding solidarity from *all* actors and institutions under EU law, not just EU citizens among each other. This includes also collective private actors, but also the member states, their governments, and the institutions of the EU.

In light of the many ongoing crises in the EU, however, solidarity now more than ever seems to be what Gallie called an “essentially contested concept,” i.e., one that “inevitably involve[s] endless disputes about [its] proper uses on the part of [its] users” (Gallie 1956, p. 169). For example, the Visegrád Group’s^4^ redefinition of solidarity in the EU as “flexible solidarity” in the refugee crisis, or the struggle regarding payments for (and from) the EU budget in light of Brexit come to mind here. But also the Eurozone crisis has shown the level of contestation between member states and EU institutions by calling into question whether solidarity mainly implies helping fellow member states in need (like Greece, Ireland, Portugal, Spain, or Cyprus) with financial assistance (such as debt cuts), or whether it requires recipients to also implement reforms and cutbacks within the public sector.

How to react to the ongoing crises and the palpable lack of a common notion of solidarity in EU politics? The strategy of Community institutions is simple and straightforward. Besides well-intentioned but limited initiatives, such as launching a ‘European Solidarity Corps’ of young people volunteering in diverse community projects and around Europe, the main approach appears to be creating rhetorical pressure by constantly calling for more solidarity among member states and EU citizens. In the past, the “strategic use of norm-based arguments” (Schimmelfennig 2001, p. 48) has proven successful in deadlocked EU politics, for example in the negotiations about the Eastern enlargement (Schimmelfennig 2003). However, this rhetorical strategy of repeating the mantra of “more European solidarity” turns out to be largely ineffective at resolving the current crises (Grimmel 2017, pp. 162–165). On the contrary, it creates a situation in which the meaning of solidarity becomes increasingly blurred, because it remains unclear how the value translates into concrete and collective practices of solidarity in the EU.

Against this background, the focus of this study will be set on member states’ politics within the EU framework, since these still seem to play the primary role in the definition and implementation of solidary actions in the EU’s approach to crisis management. The question that arises in this context and that will guide the subsequent discussion is essentially this: to what extent do the crises discourses within the institutional framework of the EU reflect solidarity, and which role do member state politics play in this context.

To approach this question, however, this article has to solve an underlying puzzle, namely to abstain from using an encyclopedic, and in this sense essentialist, notion of solidarity that just replaces the value of solidarity with other values or concepts, such as fairness or equality. I will maintain that this puzzle can be solved by a rather pragmatic approach to solidarity that starts from practices that are widely acknowledged to reflect solidarity in everyday situations (instead of essentialist and encyclopaedic definitions). The foundations of this approach to solidarity lie in the philosophical traditions of Wittgensteinian philosophy of language and (neo)pragmatism—a strain of thought that has not yet been fully developed in the studies of solidarity. Yet, as such approach claims to be both theoretically well-founded and practice-related, it could open new trajectories in this field of conceptual research of solidarity.

Building on this basis and in order to address the above mentioned question, I contend that norm-based arguments are ineffective at solving crises *not* because of weak institutionalization of the value of solidarity within the EU, or because it would be insufficiently acknowledged by member states. Rather, it is argued here that even if there is a general consensus among the central actors of the EU that solidarity lies at the heart of the integration process, the concept is regularly superseded by a host of political interests and other grounds for action that conflict with practices based on *voluntariness, selflessness* and *identification*. These practices, I claim, are widely acknowledged in everyday situations as being inseparable from the use of solidarity as a description of action, irrespective of the existence of altruistic motives or moral standards of any kind. Such a pragmatic and practice-based reading of the meaning-in-use of solidarity allows for an understanding of the concept that both goes beyond strategic interpretations and abstains from definitions of what actors ought to do to comply with standards of solidarity.

Moreover, such a practice-based reading of solidarity also contends that there cannot be different standards of solidarity that apply to different kinds of actors or institutions and that there could be a difference in what we accept as expressions of “solidary action” when it comes either to individuals, corporations, member states, or EU institutions. This places the approach not only very much in line with Schuman’s original demand of founding European integration on a common understanding and practice of solidarity. It also maintains that there already exists a baseline understanding of what solidarity means in everyday contexts of action, but that this understanding is disputed on the EU level because of other considerations, most notably interest-based politics. The major advantage of a pragmatic approach is thus that it can disclose such considerations by offering a clearer standard of practical solidarity that allows for a more critical analysis of the use of the concept of solidarity in political discourses.

It is true that other concepts are often linked to solidarity as well, such as reciprocity and fairness.^5^ Yet, other than practices of voluntariness, selflessness and identification, these are rather supplementary, but not necessary to contend that a specific act is an act of solidarity. Reciprocity and fairness may or may not be part of an act of solidarity (e. g. help victims of natural disaster) and they may be (self-declared) grounds for action that can help to convince others that *this* is an act of solidarity (e. g. take part in a student protest against the Vietnam war), but they are not indispensable in most cases of solidary action.

In a first step, this article starts by a rather philosophical discussion of how practices of voluntariness, selflessness and identification can be considered building blocks of solidary action in a number of contexts. The aim of this practice-based reading of solidarity is not to engage in an essentialist attempt to define the concept of solidarity by “hold[ing] on to the notion of ‘intrinsic, context-independent, property’” (Rorty 1991, p. 99), but to (re)describe the concept as *enacted solidarity*, by discussing how it is regularly used in political situations on the inter member state level. Such a linguistic *and* pragmatic reading of the concept claims that solidarity needs to be translated into concrete practices that are commonly acknowledged as *expressions* of solidarity within a given context, and that these practices serve as the point of reference for a common and shared understanding of the concept. In a second step, I will discusses references to solidarity in the context of two major EU crises—the Eurozone and refugee crises—to show how one or more of these elements are essentially missing in crisis management among EU institutions and member states. Finally and in a third step, I will argue that this weakness of solidarity in crisis management is based on more than the mere reluctance of member states to act in solidarity. It is related to a general contradiction between core-practices of solidarity and the EU’s current interest-based mode of integration.

## Elements of enacted solidarity: towards a language- and practice-based reading

In this section, I will argue for an approach to solidarity that starts from everyday practices and societal convention rather than from ideal–typical definitions of “what solidarity really means.” To show why this is necessary to approach solidarity in the EU and what the value-added of such a more practice-based reading is, I will draw on two contemporary and much-debated strains of thought that converge in linking “the word and the deed”: Wittgenstein as a main root of the linguistic turn on one side, and (neo)pragmatism on the other. By embracing central insights from both traditions, it will be argued that references to solidarity in the political context of the EU always have to be related to a set of regular practices that are associated with solidarity. Focusing on such meaning-in-use of solidarity is claimed here to be a most fruitful level of analysis supplementing the current discourse, since it (a) transcends the self-perception and perception by others when it comes to solidarity, and (b) allows for a more critical assessment of solidarity in the crises discourses of the EU by reference to an already existing consensus.

Underlying every conceptual as well as empirical study of solidarity, there is an essentialist temptation to find or use a definition that reflects the true and timeless meaning of the concept that captures its intrinsic properties, and its nature. Aristotle is the most prominent proponent of this idea. For him the only way to “know a thing” is “by knowing its essence.” Karl Popper criticized this view not only for its underlying “ideal of perfect and complete knowledge,” but also for its encyclopedic notion of meaning that stands in contrast with empirical sciences, because it puts “intellectual intuition, a mental or intellectual faculty which enables us to grasp the essences of things, and to know them” at the center of definitions, and more generally, of all knowledge (all quotes Popper, 1945, pp. 9–10).

The essentialist starts from the assumption, as Richard Cartwright put it, “that among the attributes of a thing some are essential, others merely accidental” (1968, p. 615) and that it is our task to separate the two. A table, for example, might be considered to consist of a number of possible properties, of which only a flat surface and four legs seem essential. Adding other attributes (like a rectangular shape, a specific color, a tablecloth, or a certain height) to the definition of ‘table’ would be considered inappropriate, since some (but not all) tables may possess these properties. In other words, these properties are not part of the *essence* of a table, but merely unnecessary embellishment. From such an essentialist viewpoint, solidarity consists of a number of essential attributes, and it is our task to discover what these are in order to develop a clear-cut, timeless definition of the concept.

The most pronounced criticism of such prototypical definitions was developed in the context of the linguistic turn, most notably in the works of Ludwig Wittgenstein and neo-pragmatist thinkers such as Richard Rorty. Although Wittgenstein and Rorty follow different arguments and arrive at different conclusions, they converge on a foundational point—the primacy of language *and* practice,^6^ i.e., the view that every explanation or definition can only manifest itself as conceptual (re)presentations of their subject matter through the practical application of linguistic tools within their specific context (Grimmel and Hellmann, 2019). At the same time, they reject the belief that language can be a “mirror of nature” (Rorty, 1979) in which “[e]very word has a meaning” that is “correlated with the word” (Wittgenstein, 2001[1953], § 1). For them, the central problem with an essentialist approach—and this also applies to the concept of solidarity—is that it maintains an artificial distinction between the conceptual and the empirical that makes us “look for an explanation where we ought to look at what happens as a ‘proto-phenomenon’,” as Wittgenstein says. “That is, where we ought to have said: this language-game is played” (Wittgenstein 2001 [1953], § 654).

Other than in any kind of essentialist reading of solidarity, Wittgenstein and pragmatists do not seek any true meaning or objective truths about the concept. Instead, they claim that there is no other access to the world other than through the *application* of linguistic tools. Based on this idea, they start from the actual, everyday language games and ordinary social practices in which a concept such as solidarity is invoked. They ask for the meanings-in-use of concepts, and see these as being part of a wider social context. For pragmatists and Wittgensteinians there is no other meaning of solidarity than that of a practice of some kind that is commonly acknowledged to reflect solidarity. Or, as Rorty (1989, p. 177) explains:

[T]here is nothing deep inside each of us, no common human nature, no built-in human solidarity, to use as a moral reference point. There is nothing to people except what has been socialized into them – their ability to use language, and thereby to exchange beliefs and desires with other people.

For such a language- and practice-based approach, it does not matter whether we think about individual or collective actors, i.e., whether we consider forms of transnational, supranational, international, intergovernmental or—more general—human solidarity (see de Witte 2015; Knodt and Tews 2017, p. 51). What matters are the actual *practices*—the expressions of solidarity that are shared among various actors and across diverse contexts of action that establish a link between these actors. In this sense, according to Rorty, “solidarity is not thought of as recognition of a core self, the human essence, in all human beings. Rather, it is thought of as the ability to see more and more traditional differences (of tribe, religion, race custom, and the like) as unimportant when compared with similarities with respect to pain and humiliation—the ability to think of people wildly different from ourselves as included in the range of ‘us’” (Rorty, 1989, p. 192).

Thus, our approach follows Wittgenstein by asking about “things we do, language games we play” and shares the pragmatists’ “emphasis on a deep layer of human action or practice that informs our experience of the world” (Goodman 2002, p. 175). The most game-changing point of this perspective, however, is that the focus shifts from the *idea of solidarity* to the actual *practice(s) of solidarity*. It is thus no longer the intellectual intuition that allows for insights into the meaning of the concept and poses serious questions when applying the concept, as described in more detail later in this article. Rather, it is about the “conceptual worlds” (Bloor 1997, p. 99) of social institutions, how solidarity is used in everyday contexts, how it works in these various contexts, and how this knowledge of the “meaning as use, meaning-in-use, or meaning as *immanent in* use” (Read 2000, p. 75, emphasis in the original) of the concept can be transferred to other, more sophisticated contexts. Wittgenstein renders this into the programmatic and well-known formula “don’t think, but look!” (Wittgenstein 2001[1953], § 66, see also § 124).

The main difference of such a language- *and* practice-based approach to any essentialist reading of the concept is that it can start from commonalities and joint interpretations that are not just reflected, but *manifested in* the regular concurrence of language and practice. Rather than engaging with abstract and chameleonic notions of solidarity or intended meanings of the concept that normally come along with a strong normative or even moral impetus (cf. Kolers 2016), we will use a comparative approach here that first assesses the everyday use of solidarity in ordinary language games and then analyzes how it is used in more sophisticated discussions of what solidarity implies in the political context of EU crises.

An in-depth analysis of the long-standing discussion between proponents of essentialist vs. anti-essentialist positions is beyond the scope of this paper. Also, I will abstain from the usual overview of the literature on the concept of solidarity and the various interpretations of the concept (for such an overview, see Grimmel 2019). I instead follow Wittgensteinian and pragmatist arguments in choosing to neglect “true meanings” in favor of solidarity as a linguistic concept, and shed light on its “practice and practical rooting” (Schatzki 1996, p. 136). It is thus not intended to say anything about “real motives” (or interests; see, e.g., Hechter 1988) that may, or not, lie at the bottom of any solidary action or to engage in the conceptual history of the solidarity concept (for such a study, see, e.g., Stjernø 2009), but to argue that solidarity is part of a specific yet contingent practice that is based on certain grounds for action commonly shared within the boundaries of a distinct context of action. In other words, the reasons actors provide as grounds for their action do not necessarily have to match their motives or interests and could still be accepted by others as practices of solidarity.

My three main goals are (1) to probe the value of such a theoretical perspective in an empirical case study, (2) to show how this allows a critical perspective on the roots of the EU’s insufficient crisis management, and (3) to understand how solidarity could play a more fruitful role in European integration. Against this backdrop, I start by describing three grounds for action and by arguing that these build the basis for enacted solidarity in various contexts: *voluntariness, selflessness* and *identification*. These elements are explicitly not to be understood as constituting a timeless, irreducible, or even a complete definition of what solidarity *is*. Rather, it is claimed here that these are elements of practice that are present in most everyday situations that are regularly described to reflect solidarity and on which actors can rely in their actions. As such, they provide grounds for action and can be considered a point of reference for practices of solidarity in the political sphere and among diverse groups of actors. Deviations from these practices, it is maintained here, would at least need justification by actors why these elements are not applicable in a specific situation, or why there should be a different standard of solidarity to be applied. This view does not entail a rigid understanding of solidarity, in the sense that it is only reflected in situations in which nothing but practices of voluntariness, selflessness and identification have a place. The standard cases are certainly situations in which a number of motives, reasons and rules come together. The point is rather that it is hardly possible to think of solidarity without these three practices playing a central role in a specific situation.

### Voluntariness

Solidarity is a delicate value to apply in practice. It is easily contested by attempts to enforce it, either by physical force (which is a rather extreme possibility) or any kind of external pressure or legal obligation that conflicts with the idea of the free will of the actor. Acting in solidarity is generally based on the absence of coercion from others. After a natural disaster, for example, state A may appeal to the solidarity of state B by asking for help (such as engaging in rescue missions or providing humanitarian aid), and it can even demand the support of its neighbor state by reminding it of their close ties. And even though state B might have been reluctant to support state A, its decision to do so is still an expression of solidarity because it was made voluntarily. However, if one state tries to threaten another to make it help, for example by insinuating consequences for future political or economic relations, it is hardly possible to tell whether state B’s decision to help was based on a voluntary decision or on state A’s threat. However, the result is the same: Analogous to the impossibility of forcing someone to be free, in the various language games in which solidarity plays a role, it seems to be rather odd or even contradictory to invoke the concept as an enforceable practice. In other words, the meaning-in-use of solidarity is closely linked to actions that are explicitly or implicitly expressions of the idea of actors’ freedom of choice. An exception, of course, is that an actor might feel obliged to do something, which still depends on the actors own appraisal of the situation and his or her sense of being bound to perform certain duties.

### Selflessness

It is possible to imagine a case in which an actor acts voluntarily and refers to the value of solidarity, but the actual grounds or reasons for action are not related to solidarity. An actor might, for example, have a self-interest in acting, and it might be a rational choice to camouflage the true considerations behind a solidarity-based justification. In such a case, alter could help ego just to fulfill his or her own private aims, or in anticipation of a later payoff. Borrowing ego money to implement a social project on the grounds of expecting high returns is not exactly an expression of alter’s solidarity towards ego and the project. The actor would follow a rule, to use Wittgenstein’s logic, but this would not be an expression of the concept of solidarity. What is missing is selflessness understood as the absence of other, private, motives. This does not make every act of solidarity an act of charity. And it does certainly not preclude that we understand solidarity as a value that demands at least a minimum of reciprocity (in that you can expect that others would be solidary with you in a similar situation) (Flache and Macy 2006) and mutual trust when showing solidarity with somebody (Taylor 2015, pp. 136–139; Hilpold 2015, p. 258). It instead creates demands on the grounds for action that go beyond the mere voluntariness of the actor and call for other rules for action than those based on self-interested motives.

From our perspective, this is also, and explicitly, not a question of the motivation or interest of actors—i.e., the will and belief to engage in such practices—because a belief in following a rule or principle such as solidarity does not necessarily mean that the rule or principle is actually followed. Wittgenstein made this clear in his famous “private language argument” by stating that “to think one is obeying a rule is not to obey a rule. Hence it is not possible to obey a rule ‘privately:’ otherwise thinking one was obeying a rule would be the same thing as obeying it” (Wittgenstein, 2001[1953], § 202). Put another way, to think one is acting *in accordance with* the principle of solidarity is not necessarily the same as acting *in a way that reflects* solidarity. This brings us to a last ground for action that builds the basis for practices of solidarity in various contexts.

### Identification

From a Wittgensteinian as well as from a pragmatist perspective, there is a general need for perceptible expressions of every value, principle, rule, concept, or any other linguistic category to define its concrete meaning. Embedded in a social world, such perceptible expressions occur in the form of practice, i.e., the regular application of a rule or concept in concrete cases. Wittgenstein put it into the formula that every “inner process stands in need of outward criteria” (Wittgenstein 2001[1953], § 580); pragmatists called this the “primacy of practice” (Putnam 1994, p. 152). Only by practicing linguistic expressions and attaching meaning to them “as we go along” does the constant comparison and mutual correction of the meaning-in-use of concepts and rules become possible. This makes the definition of meaning no longer a domain of “private” reflection, but an essentially social endeavor, since, as Kripke pointed out, actors are normally “interacting with a wider community” that will necessarily react to divergences in interpretation or application that are manifested in practice. “Others will then have justification conditions for attributing correct or incorrect rule following to the subject, and these will not be simply that the subject’s own authority is unconditionally to be accepted” (Kripke 1982, p. 89).

This “practice and practical rooting” (Schatzki 1996, p. 136) of “conceptual worlds” (Bloor 1997, p. 99) within social institutions cannot be overestimated, since it reveals a central weakness of solidarity as a core value vis-à-vis other core values, such as democracy (Niżnik 2011), in the EU today. I discuss this point in detail below. For now, it is important to understand that linguistic practices fulfill a function that goes beyond the mere physical act of “doing something.” Linguistic practices are social acts of ascertainment and adjustment of meaning as well, which also means that individual actors cannot decide alone what a concept like solidarity means in practice, or when they act in accordance with the concept.

This also applies to the expressions of enacted solidarity that we have identified in practices that reflect voluntariness and selflessness and to a final element that is commonly associated with the concept and practice of solidarity—the identification with either others or a shared idea. In this sense, solidarity also comprises practices in which the association or linking of one’s own preferences, views or beliefs seems to match those of others. One could identify, for example, with the labor movement, the idea of sustainable ecological development, the fight against racism, or a group of people suffering from a civil war. In these cases, identifying oneself with a case, person, or group is much more expressive than acting based on voluntariness or selflessness. Practices of identification showcase a certain feeling of belonging or affiliation that is typically part of a larger narrative and “crucial to the construction of affinity groups” (Dean 1998, p. 14) that can serve various social functions, such as creating or reassuring a sense of togetherness.

In this sense, identification is often closely linked to reciprocity or reciprocal expectations as an often-adduced element of solidarity (see e. g. Eschweiler et al. 2019). Normally, we expect that others are at least willing to help us when we help them. Yet, in fact, there are also cases where we cannot reasonably expect those suffering to help us one day later, e. g. because of differences in the potential to help. Moreover, in the end, we can just hope that others are willing to help us in return. At least, it would hardly be an act of solidarity if we would understand it to be a tit-for-tat game in which ensured reciprocity is a precondition for helping others.

The expressive identification of enacted solidarity is thus crucial since it involves relating to others, an idea or objective that goes beyond the concept itself. Practices of identification give solidarity a concrete aim or objective that is necessary for action. As such, they serve as a necessary specification of the concept, and may provide reasons or justifications for action.

## Solidarity in times of crisis

The previous section has shown how a practice-based reading of solidarity can avoid an essentialist definition of solidarity that is present in most political discussions, and even a major part of the scientific literature on solidarity in the context of the EU. It has been shown that such definition builds on an artificial distinction between the conceptual and the empirical, and that a practice-based concept of enacted solidarity could be a fruitful supplement to these debates in that it asks for how solidarity translates into concrete action. Based on this theoretical background, this section discusses the concept of solidarity in the empirical context of two major EU crises of the last decade: the Eurozone and the refugee crises. Here, it focuses on member states’ uses of solidarity in political discussions within the EU. A central aspect of this study will be that it intentionally refuses the claim that there could be different standards of solidarity on the various levels of action and between various groups of actors within the framework of the EU. Based on a practice-based reading of the concept outlined in the previous section, this analysis does not seek to identify mere references to solidarity in political declarations, claims and common action plans. Rather, it critically assesses instances in which the EU and its member states invoked the solidarity concept, and examines the extent to which these have been connected to practices of voluntariness, selflessness and identification.

### The eurozone crisis

More than ten years after its outbreak, the Eurozone crisis has developed into a permanent problem of the EU. Even though the EU has taken a number of ambitious measures—such as the establishment of the Single Resolution Mechanism or conducting stress tests on financial institutions—and media attention in the member states has faded, there seems to be a broad consensus that the crisis is far from over, and that more joint efforts are required to prevent another outbreak. In addition to the political measures realized, such the European Stability Mechanism or the Outright Monetary Transactions of the European Central Bank, a recurring pattern in the discourse about sustainable economic reforms, recovery proposals and long-term solutions is the call for greater solidarity among member states. These calls have often gone unheard, since the idea of what solidarity implies in practice remains highly divergent. The struggle between Germany and Greece over the back-payment of debts, possible debt cuts and the implementation of reforms is an example of such divergence.

One the one hand, the former Greek Prime Minister, Alexis Tsipras, had claimed that austerity measures are incommensurate with solidarity by acknowledging that “lending is certainly a form of solidarity”^7^ while at the same time accusing the EU of attempting “to suffocate [the Greek] people.”^8^ Therefore, he argued, these measures have to be considered “unacceptable in a Europe of solidarity and self-respect.”^9^ On the other hand, the German government and especially Chancellor Angela Merkel emphasized that Greece already experienced an “unprecedented amount of European solidarity,” but also that “solidarity and responsibility go hand in hand”^10^ and that “solidarity has to be coupled with individual responsibility, with reforms in those countries that we are helping.”^11^

These two well-known positions, which are shared by a number of other proponents as well, reflect not just a divergence in perceptions of what the EU’s fundamental value of solidarity implies in light of the debt crisis for creditor and debtor states. Even more telling is the obvious instrumentality and strategic use of the concept to promote a particular political position and to create rhetorical pressure. This strategic appeal to solidarity can also be found in more recent statements, like that of the former head of the Eurogroup, Jeroen Dijsselbloem, who has asserted that “[d]uring the crisis of the euro, the countries of the north have shown solidarity with countries affected by the crisis,”^12^ but that these countries “also have obligations.” French President Emmanuel Macron also associated the concept of solidarity with a political program, namely his vision of deeper European integration that includes tasks and duties by concluding that “[e]veryone needs to reform but that implies solidarity.”^13^

If we consider this situation in light of our three-part practice-based reading of solidarity, the practices of voluntariness, selflessness and identification do not play a considerable role here. Rather, mutual claims and outstanding debts—either in monetary form or in the shape of reform and austerity measures—were the main categories of the debate. Solidarity obviously was not much more than the dependent variable of political interests and their enforcement. This conditionality of solidarity upon the fulfillment of other—political and economic—aims was most paradigmatically summarized in the words of the former president of the European Commission, José Manuel Barroso, who stated that “[t]here is no stability without solidarity and no solidarity without stability.”

All this does not preclude that member states and EU institutions were keen to portray their own actions as selfless (bailout programs), and the obligations of the other side (steps taken to reduce deficits) as a voluntary choice, i.e., as expression of solidarity. The point is rather that the practice was another one, reflected a rational cost–benefit analysis and involved a series of “classical” bargaining rounds based on national interests. After the dimension of the debt crisis was obvious and Greece, Ireland, Portugal, Spain, and Cyprus announced their need of bailout funds, it was simply not a question of choice anymore, but a clear economic necessity coupled with the strict specifications of the European Fiscal Compact (2013). These specifications included far-reaching provisions to be transposed into national legal orders, such as a balanced budget rule, an automatic correction mechanism, and a monitoring institution in order to enforce the “debt brake” criteria outlined in the Stability and Growth Pact (SGP 1998) and its reforms in the “sixpack” (2011).

In such a setup, which was largely dominated by language games of balancing political interests, there was simply little room to make voluntary or selfless choices. The same is true for practices of identification with common aims (such as the social welfare and prosperity of *all* member states) and the problems of others (mainly insufficiencies in the organization of the public sector and a lack of economic competitiveness), which faded away in light of the clear-cut distinction between creditor and debtor states. This led to a somewhat paradoxical situation in which the increase in appeals to solidarity was opposed by political and economic considerations that were based on practices of strategic cost–benefit calculations and political bargaining. We can observe a similar pattern in the reactions to the EU’s refugee and migrant crisis.

### The Refugee and migrant crisis

The refugee and migrant crisis has been described as the most palpable expression of the lack of solidarity within the EU (cf. Knodt and Tews 2017, pp. 59–61). Critical observers have pointed out that “[t]he increased number of migrants coming to Europe is obviously a crisis for the migrants who are travelling, but it can barely be seen as a migration crisis in Europe. As the European countries have resources to handle the number of migrants, the crisis in Europe is rather related to the many different reactions. The conflicting ideas of solidarity have laid premises for these” (Takle 2018, p. 127; see also Bell 2010). Indeed, the refugee and migrant crisis has clearly exposed the flaws of the Dublin System and the inadequate attempts to reform it such as the Dublin III Regulation (No. 604/2013). It has also revealed the manifold different interpretations of solidarity in light of crisis, and member states’ unwillingness to engage in intergovernmental solidarity beyond what is currently legally required in the EU. Considering the rules of the Dublin System, however, there seem to be few good reasons to believe that it constitutes a fair system, since it leaves the main financial and administrative burdens in the hands of member states that have external borders of the EU, like Greece, Spain, Italy, and Hungary.

When the Dublin Convention was signed in 1990, the situation was certainly different than in light of the developments since 2015, when rising numbers of people arrived in the EU. But it is still the rule that the state of first entry is responsible for examining an asylum application, and all attempts to establish a more common system have failed so far. In other words, the EU is stuck in a political construction that might have worked fine a quarter of a century ago, but it has proven to be a largely inappropriate system that does not address the current challenges. This situation brings to mind what Fritz W. Scharpf described as the “joint decision trap” in federal and quasi-federal political systems:

[N]on-agreement is likely to assure the continuation of existing common policies […]. In a dynamic environment, the implications for the substantive quality of public policy are obvious: when circumstances change, existing policies are likely to become sub-optimal even by their own original criteria. Under the unanimity rule, however, they cannot be abolished or changed as long as they are still preferred by even a single member.[…] That also means, however, that member governments will be precluded from dealing individually with pressing problems even if the Community cannot agree on an effective solution. In short, joint-decision systems are doubly vulnerable to the consequences of non-agreement: they may be incapable of reaching effective agreement, and they may lose the independent capabilities for action of their member governments (Scharpf 1988, p. 257).

Even though the hurdles are not that high and a qualified majority in the Council of the EU would be sufficient to change the Dublin System, it is unclear whether it will be possible to implement necessary reforms against the will of the member states. At the same time, states are bound to the common rules, even though they lead to sub-optimal political outcomes or even a capacity overload in single member states. This was already the case before the refugee and migrant crisis, when states without external borders largely neglected the situation on the Italian island of Lampedusa since the early 2000s and after the Arab Spring of 2011, or on the Greek islands of Crete and Rhodes after the outbreak of the Syrian Civil War in 2012. And it is still the case when, for example, the states of the Visegrád Group (the Czech Republic, Hungary, Poland and Slovakia) criticized the proposal to reform the Dublin System for its “mandatory system of redistribution of asylum seekers” by arguing that it “could act as a pull factor and will further divide Member States (and their respective societies).” For this group of states the proposal of “the European Commission … on the reform of the Dublin system” simply fails “to enjoy unanimous support by the Member States.”^14^

In terms of solidarity, this situation inevitably results in a dilemma: whether or not a reform decision is reached, it will create constraints on either the reformers’ side or those in favor of “flexible solidarity” (with no mandatory quotas, but voluntary contributions based on what member states are willing to offer).^15^ In both cases, however, such agreement or non-agreement can hardly be grounds for solidarity, since it precludes voluntary commitments—either on one or the other side. It is a political decision that is reached in a joint-decision system in which interest-based considerations are still the dominant basis for decision-making.

The epigraph by the former President of the EU Commission, Jean-Claude Juncker, at the beginning of this article is particularly telling given the fact that—65 years after solidarity was codified in the Preamble to the European Coal and Steel Community Treaty—none of the earlier defined practices of solidarity played a significant role in the EU’s crisis management. At least, I see no stage or field in which voluntariness (e.g., engaging in a fair financial or administrative sharing of burdens), selflessness (e.g., by disinterestedly discussing how to replace the outdated Dublin system), or identification with the challenges, problems and troubles of others (e.g., those of asylum seekers, or member states with external EU borders) played a significant role in this highly politicized and legalized field of the EU. And attempts to enforce such practices will miss their mark, since, as Martin Nettesheim asserts, “[e]nforced ‘solidarity’ is not solidarity but constraint.”^16^ This does not mean that solidarity *should* not play a more active role in EU migration policy. Rather, the point is that it *can not* in the context of the current deadlocked political situation and an EU asylum system that was created in different historical circumstances, that has remained artificial, and that has been upheld because some member states have profited from it at the expense of others.

## Enacted Solidarity in the politics of European integration

The starting point of our discussion was an understanding of the concept of ‘solidarity as enacted solidarity’ that attaches equal importance to language *and* practice. Building on long-standing criticism of an essentialist reading of concepts, this analysis of EU crises has been based on a Wittgensteinian as well as pragmatist reading of the concept that started from the claim that both language *and* practice have to come together in order to develop a meaningful solidarity concept and allow for a common understanding of what solidarity implies. What is most striking from this vantage point is the divide between the frequent use of and appeal to solidarity in crisis situations on the one hand, and the lack of practices reflecting solidarity on the other hand. In other words, even though “’human solidarity’ has emerged as a powerful piece of rhetoric” (Rorty 1989, p. 192) in western societies, it has rarely been applied to common practices of solidarity in the EU (at least in the cases we examined in this study). This brings us to the central question: why this is so? Why does solidarity as a core value of the EU not play a more vital role in the politics of European integration when it is most needed—in times of crisis?

Politics provides an easy answer to this question: It is the lack of solidarity itself, the unwillingness of member states to engage in “real solidarity.”^17^ But it is not that easy. It would fall short to appeal to a greater sense of community among member state governments, because the willingness or unwillingness to show more solidarity very much depends on the context of action and the language games that are played in this context. There might be contexts in which individuals, groups, or states are more willing to show solidarity than others, namely those in which voluntariness, selflessness *and* identification (with others or a common idea) are part of the rules of the game. Our analysis of the Eurozone as well as the refugee and migrant crisis, however, has shown that there are few incentives for such practices; instead, EU member states tend to engage in a cost–benefit analysis of different reactions to the problems at hand. This does not preclude a common definition of the problem, of course. It rather suggests the existence of specific reflexes and trajectories of how to cope with problematic situations and crises.

In the EU, these reflexes and trajectories are shaped by a specific mode of integration and a regulative idea that could best be described as an ‘interest paradigm’. I have examined the roots and consequences of the interest paradigm of EU integration in detail elsewhere (Grimmel 2018). It should be sufficient here to stress that the founding states of the Treaty of Paris (1951) and the Treaty of Rome (1957) chose a path of integration that favored small steps of economic integration (that would be eventually followed by political integration) over a federal grand design. Faced with the political realities of the early days of integration, there was no other option than engaging in what Jean Monnet and Robert Schuman thought of as a step-by-step approach of European unification.

But the decision to base the integration process—at least in part—on economic considerations came with a price. In retrospect, this decision was a critical juncture, since the idea underlying this approach set the course for the politics of the EU today by introducing the idea that the integration process must offer a “multitude of different advantages to different groups” (Haas 1958, p. xxxiii) of actors to ensure their continuing acceptance. The side effect of this decision was momentous: from that point on, the further course of integration significantly depended on actors’ interests and the benefits they expect from participating in the European project. The EU and its predecessors constantly had to justify themselves in terms of the benefits they provided for the member states. The EU never left this path chosen in the early days, and the mode of integration is still essentially based on the idea that purpose-rational *homines oeconomici* (must) foster European integration by following their self-interests.

The scholarly debates of integration theory have evidenced this fact by the broad consensus that the ‘pursuit of interests’ has to be seen as a major driving force of integration. Actors’ individual and corporate interests, and their rational ways of enforcing them, are understood here as the driving forces or the “motor” behind the European integration process. The main dividing line in these classical approaches seems to be the question which actors and institutions on domestic, transnational, national and supranational levels have to be considered most influential and formative. This is at least the case in the main descriptive-analytical schools of thought such as (neo)functionalism, (liberal) intergovernmentalism, supranational institutionalism, and also parts of multilevel governance approaches (for overviews of these various actor-centered theories, see, e.g., Rosamond 2000; Wiener and Diez 2009; Grimmel and Jakobeit 2009; Nelsen and Stubb 2014).

Our original question was “to what extent the crises-discourses within the institutional framework of the EU reflect solidarity, and which role member state politics play in this context?” If we agree that an ‘interest-based mode of integration’ centered around national politics has been the dominant modus operandi of EU politics, we see why the reflexes and trajectories of how to cope with crisis situations do not turn out to be more solidary, and why even the “strategic use of norm-based arguments” (Schimmelfennig 2001, p. 48) has proven ineffective.

Admittedly, by focusing on member state politics, this article can only draw an incomplete picture of solidarity practices within the framework of the EU. The insights of this study, however, can be transferred to practices of solidarity beyond national politics and unfolding transnationally at least insofar as such practices constitute primarily ‘horizontal’ relationships between individual actors. Yet, EU member states are obviously still crucial actors when it comes to crisis management and practices of solidarity—as the Eurozone and refugee crises show.

Such practices, of course, are not in human nature, any more than there is any built-in concept of solidarity. Hollis and Smith (1990, p. 188) once said that “rationality is not a universal capacity for calculating the costs and benefits of actions which contribute to an outcome, but the applying of a local rule which supplies reasons for acting.” Indeed, we have to understand that these very local rules—i.e., those of the interest paradigm—are the reason why practices of voluntariness, selflessness and identification are not considered rules for action in times of crisis. Otherwise, we will cherish the illusion that solidarity can be stimulated within the current framework of EU politics by appealing to it—including the disappointment facing the EU’s political realities.

## Conclusion

There is, as Malcolm Ross has pointed out, “a tendency to use solidarity for rhetorical effect” (Ross 2010, p. 23). Looking at the manifold uses of solidarity in the crises of the EU, it is interesting to see how this strategic invocation of solidarity by the member states and EU institutions has not, in fact, underpinned the concept, but rather made it a weak point of reference and a floating signifier that seems to be interpreted by political actors on a case-by-case basis depending on member states’ interests rather than serving as a value and principle guiding common action. In other words, the mere increase in the use of the word ‘solidarity’ in the current crisis discourses may not be equated with a rise in solidarity in the politics of the EU, but gives rise to the question why the EU’s rhetorical pressure not translates into more significant practices of solidarity.

The point of this article is not first and foremost to make a strong (normative) claim for more solidarity, or to outline an ethical/moral definition of solidarity. It has instead been the aim to show how the “strategic use of norm-based arguments” by the members states and EU institutions has created a situation in which, in fact, solidarity is not consolidating but can only play a marginal role, because it leaves little room for its enactment in practices that are regularly associated with solidarity in everyday life, namely practices that reflect voluntariness, selflessness and identification.

Crisis situations, it has been argued here, are an important litmus test for the meaning-in-use of solidarity in the EU, because they mark times in which enacted solidarity is most needed. Considering the Eurozone and the refugee and migrant crises, *common* practices of solidarity are hardly detectable, and it seem unlikely that they will develop in the context of the ongoing politics of European integration. Thus, hopes that practices of solidarity in the European Union context will develop either as a side effect of member state self-interest, or by rhetoric pressure cannot be supported by this study.

## Footnotes

1 COM(2016) 942, Brussels, 7.12.2016, p. 2.

2 Speech of the President of the European Commission, State of the Union conference: Building a people’s Europe, European University Institute, Florence, 5 May 2017, available online: https://europa.eu/rapid/press-release_SPEECH-17-1238_fr.htm.

3 The Schuman Declaration on the creation of a European Coal and Steel Community, Paris, 9 May 1950, available online: https://europa.eu/european-union/about-eu/symbols/europe-day/schuman-declaration_en.

4 This group consists of the Czech Republic, Hungary, Poland and Slovakia.

5 See e. g. European Commission (2019) *Solidarity in Europe—Alive and Active* (paper prepared by Malcolm Ross, commissioned by the REA, coordinated by Andreas Obermaier-Mureşan), Brussels, p. 6; available online: https://ec.europa.eu/research/social-sciences/pdf/policy_reviews/solidarity_in_europe.pdf.

6 This point is shared by many other contemporary philosophers who study the linguistic turn (see, e.g., Putnam 1995; Goodman 2002).

7 Summary Comments at the European Parliament, Strasbourg, 8 July 2015, available online: https://www.primeminister.gov.gr/english/2015/07/08/prime-minister-alexis-tsipras-summary-comments-at-the-european-parliament/.

8 *BBC News*, “Greek Debt Crisis: Why a Bailout Referendum?”, 3 July 2015, available online: https://www.bbc.com/news/world-europe-33314442.

9 Ibid.

10 *DW*, “Solidarity and responsibility go ‘hand in hand,’ Merkel tells Greece”, 18 June 2015, available online: https://www.dw.com/en/solidarity-and-responsibility-go-hand-in-hand-merkel-tells-greece/a-18523981.

11 *BBC News*, “Eurozone Crisis Colours German Election Campaign”, 30 August 2013, available online: https://www.bbc.com/news/world-europe-23901299.

12 *The Telegraph*, “Anger after Head of Eurozone Finance Ministers Says Southern Europe Blew Cash on ‘Drinks and Women’”, 21 March 2017, available online: https://www.telegraph.co.uk/news/2017/03/21/anger-head-eurozone-finance-ministers-says-southern-europe-blew/.

13 *Financial Times*, “Emmanuel Macron makes case for ‘ambitious’ overhaul of Eurozone”, 6 September 2017, available online: https://www.ft.com/content/793d1d4c-92d2-11e7-bdfa-eda243196c2c.

14 Joint Declaration of the Visegrád Group Prime Ministers, meeting in Prague, 8 June 2016, available online: https://www.visegradgroup.eu/documents/official-statements/joint-declaration-of-the-160609.

15 Joint Statement of the Heads of Governments of the V4 Countries, Bratislava, 16 September 2016, available online: https://www.vlada.cz/en/media-centrum/aktualne/joint-statement-of-the-heads-of-governments-of-the-v4-countries--bratislava--16-september-2016-148913/.

16 Martin Nettesheim, „Das EU-Recht in der Krise – ein schwieriges Verhältnis “, *Verfassungsblog.de*, 15 September 2017, available online: https://verfassungsblog.de/das-eu-recht-in-der-krise-ein-schwieriges-verhaeltnis/.

17 See, e.g., European Commission, “Press release, Commission makes progress on a European Agenda on Migration”, 4 March 2015, available online: https://europa.eu/rapid/press-release_IP-15-4545_en.htm; RFI, “French MEP calls for solidarity as UK slams EU asylum quota plan”, 13 May 2015, available online: https://en.rfi.fr/europe/20150513-french-mep-calls-solidarity-uk-slams-eu-asylum-quota-plan; *Die Linke*, “Lack of solidarity continues to undermine EU migration policy”, 27 March 2017, available online: https://www.dielinke-europa.eu/de/article/11166.lack-of-solidarity-continues-to-undermine-eu-migration-policy.html.
